# Supplementary material for: Exosome-Modified Liposomes Targeted Delivery of Thalidomide to Regulate Treg Cells for Antitumor Immunotherapy
Source: Pharmaceutics. 2023 Mar 27;15(4):1074. doi: 10.3390/pharmaceutics15041074 (PMC10142880; doi:10.3390/pharmaceutics15041074)
Supplement: Supplementary file 1 [file pharmaceutics-15-01074-s001.zip › pharmaceutics-2201844-supplementary.pdf]

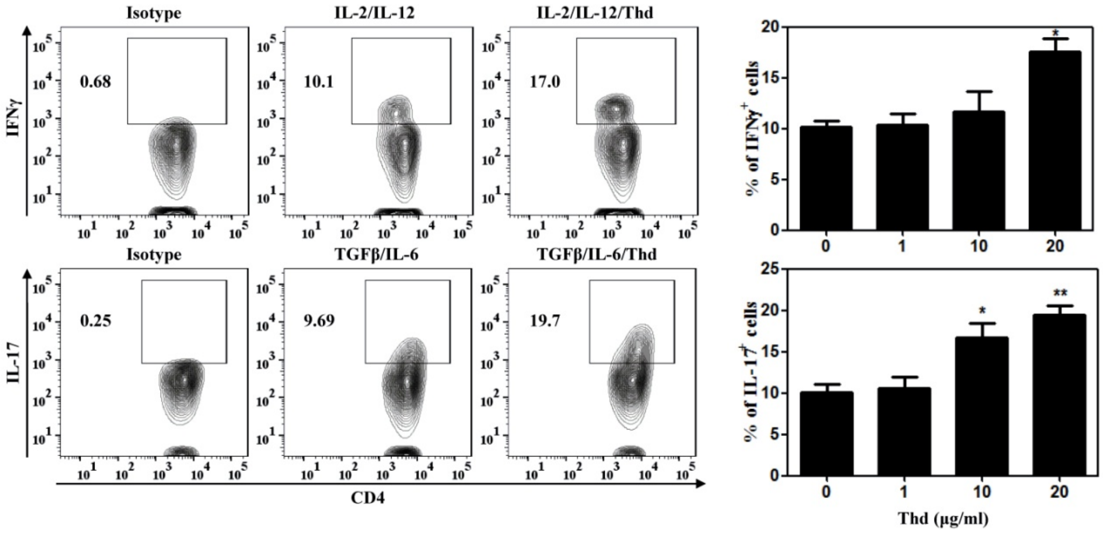

Figure S1. Effect of THD on the differentiation of Th1 and Th17 cells *in vitro*. FACS-sorted naïve $CD4^{+}CD25^{-}Foxp3/gfp^{-}$ T cells were cultured under Th1- and Th17-polarizing conditions in the presence of THD (1-20 μg/mL) for 72 hours. The phenotype of Th subsets was analyzed by FACS. Typical FACS data are shown on left panel. The right panel show summarized data (means ± SEM), pooled from 3 to 4 separate experiments (n = 9~12). *p<0.05, ** p<0.01, as compared with medium control (without THD).

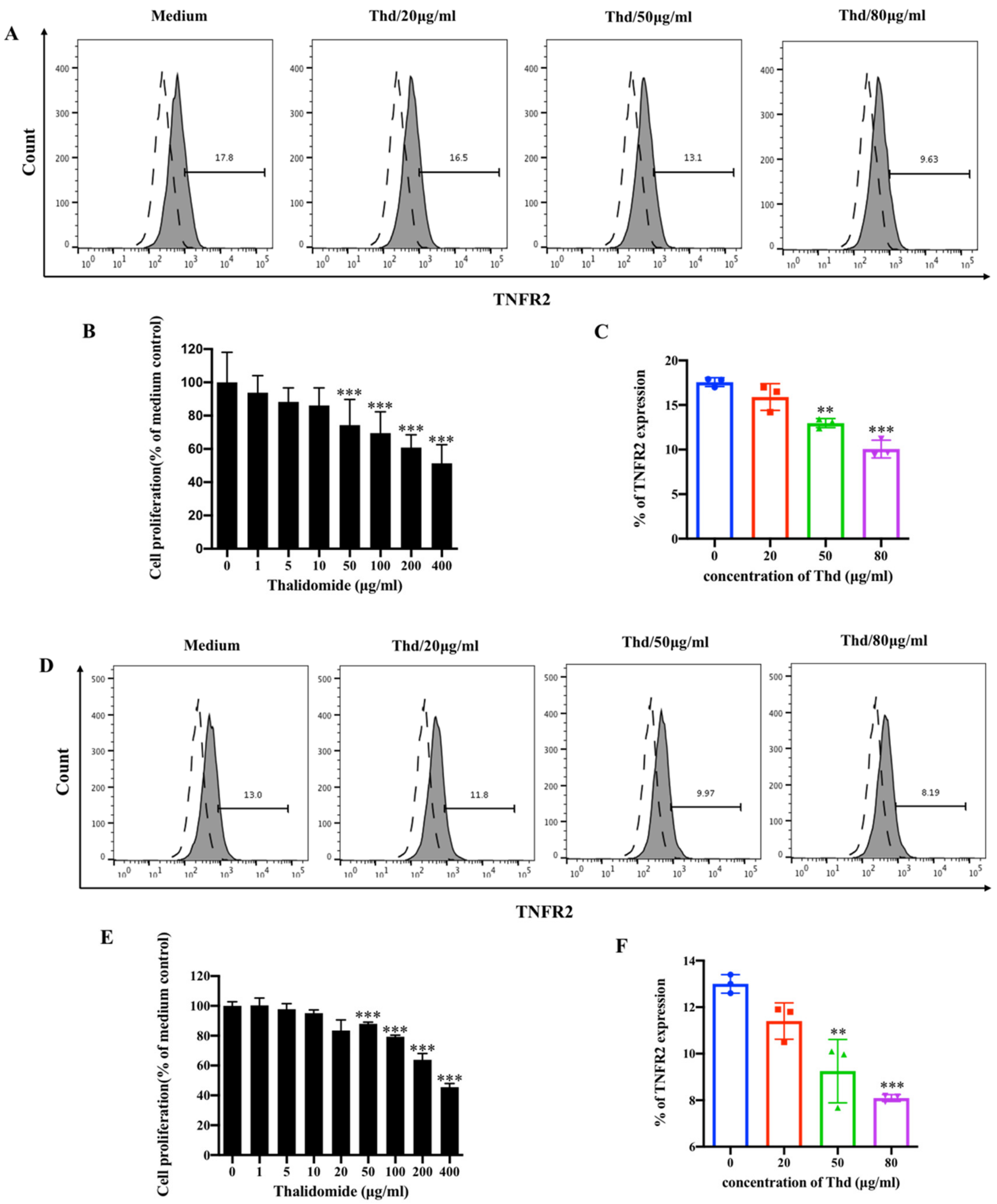

Figure S2. THD reduces TNFR2 expression on tumor cells *in vitro*. For the proliferation assay, 4T1 and CT26 cells (5×10^4/mL) were plated in 24-well flat-bottom plates. THD with a concentration range of 1~400 μg/mL were added after 12 h. After 24 h, the proliferation of cells was assessed with MTT assay. The percentage of proliferation was calculated based on the medium culture group. For the TNFR2 expression assay, 4T1 and CT26 cells (8×10^4/mL) were plated in 24-well plates, three different concentrations of THD were added and flow cytometry were performed for TNFR2 expression assay after 24 h. (A) Typical Flow cytometry data of TNFR2 expression on 4T1 cells. (B) Effect of THD on the proliferation of 4T1 cells. (C) Summarized data of TNFR2 expression on 4T1 cells. (D) Typical Flow cytometry data of TNFR2 expression on CT26 cells. (E) Effect of THD on the proliferation of CT26 cells. (F) Summarized data of TNFR2 expression on CT26 cells. (A, C, D, F) data

(means ± SEM, n = 9), pooled from 2 or 3 separate experiments with similar results. **p<0.01, ***p<0.001, as compared with medium control group (without THD). (B, E) data (means ± SEM, n = 12), pooled from 2 or 3 separate experiments with similar results. ***p<0.001, as compared with medium control (without THD).

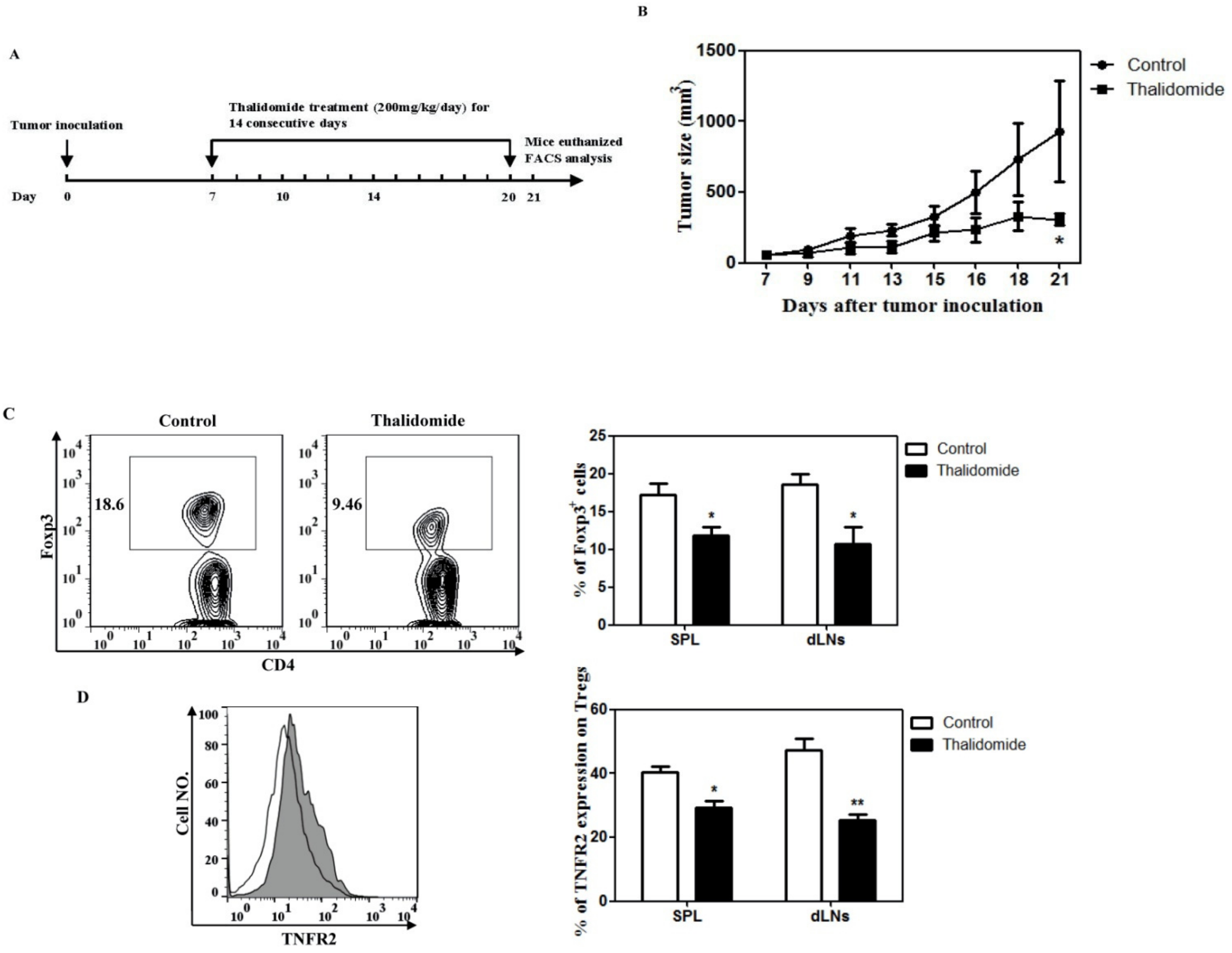

Figure S3. Effect of thalidomide on 4T1 murine breast cancer model. (A) The schematic diagram of experiment procedure. 4T1 tumor cells in single-cell suspension were injected subcutaneously with 2 × 10^5 cells in 0.1 mL of PBS into the right posterior flank of Balb/c mice. Seven days post-tumor cell inoculation, some mice were i.p. injected for 14 consecutive days with THD (200 mg/kg/day). Mice were sacrificed after 24 hours of last treatment. Spleens (SPLs) and lymph nodes (LNs) were harvested. FACS was used to analyze the proportion of Tregs in CD4 T cells and surface TNFR2 expression in Tregs. (B) Tumor volume. (C) Foxp3 expression in CD4 T cells. (D) TNFR2 expression by $Foxp3^+$ Tregs. (C, D) Representative FACS plots are shown on the left panel. (D) Gray-filled histogram: control; black solid line: thalidomide treatment. The right panel of (C, D) show summarized data (n = 9, means ± SEM), pooled from 3 separate experiments with similar results. *p < 0.05, **p < 0.01, as compared with control group.

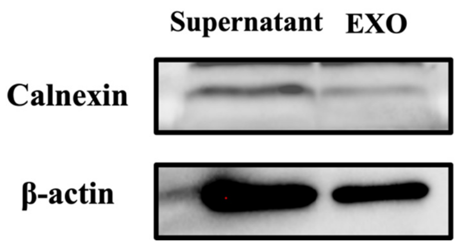

Figure S4. Validation of exosome purity. Western blot assay for the identification of endoplasmic reticulum marker protein in exosome. Supernatant was used as positive control. β-actin was used as a positive control.
